# IgG-Complexed Adenoviruses Induce Human Plasmacytoid Dendritic Cell Activation and Apoptosis

**DOI:** 10.3390/v13091699

**Published:** 2021-08-27

**Authors:** Thi Thu Phuong Tran, Tuan Hiep Tran, Eric J. Kremer

**Affiliations:** 1 Institut de Génétique Moléculaire de Montpellier, Université de Montpellier, CNRS, 34090 Montpellier, France; tranphuongdkh@gmail.com (T.T.P.T.); tuanhiepdkh@gmail.com (T.H.T.); 2 Department of Life Sciences, University of Science and Technology of Hanoi Vietnam Academy of Science and Technology, Hanoi 100000, Vietnam; 3 Faculty of Pharmacy, PHENIKAA University, Hanoi 12116, Vietnam; 4 PHENIKAA Research and Technology Institute (PRATI), A&A Green Phoenix Group JSC, Hanoi 11313, Vietnam

**Keywords:** adenovirus, antibodies, immune complexes, plasmacytoid dendritic cells, innate immunity

## Abstract

Following repeat exposure to many human adenoviruses (HAdVs), most adults harbour long-lived B- and T-cell responses. Combined, this response typically protects us for years from re-infection by the same HAdV type. In spite of these immune responses, some HAdV types are associated with persistent infections that constitute a life-threatening risk when an individual’s T-cell response is compromised. By contrast, patients with B-cell deficiencies do not appear to be at a greater risk of HAdV disease. This dichotomy begs the question of the secondary role of anti-HAdV antibodies during host defence. In this study, we explored IgG-complexed (IC)-HAdV5 and primary human plasmacytoid dendritic cell (pDC) interactions. We found that IC-HAdV5 are efficiently internalized in pDCs, stimulate their activation through TLR9 signalling, and cause apoptosis. These data may help reconcile the enigma of robust immune response to HAdVs, while concurrently allowing persistence.

## 1. Introduction

Plasmacytoid dendritic cells (pDCs) are a subset of phagocytes continuously produced in the bone marrow and, via the peripheral blood, are recruited to breaches in tissue homeostasis [1]. During a response to viral infections, the hallmark of pDCs is their ability to secrete relatively high levels of type 1 interferons (IFN-1). Combined with the innate immune responses from infected cells and other recruited immune cells, the cytokine profile of pDCs biases the adaptive immune response toward an antiviral Th1 response [2]. 

Members of the family *Adenoviridae* have nonenveloped icosahedral particles of approximately 90 nm, containing a double-stranded linear DNA genome of 36,000 ± 7000 bp [3]. They are found on every continent and now include more than 300 types isolated from mammals, fish, birds, and reptiles. Human adenoviruses (HAdVs) belong to the genus *Mastadenoviridae* and are grouped into seven species (A–G), which include approximately 100 types that are grouped by serology and/or sequence phylogeny [4]. Epidemiological data generated during the last six decades suggest that greater than 90% of us have been infected by a handful of HAdV types by the time we are a few years old [5]. Most HAdV infections cause type-specific, asymptomatic to self-limiting disease, in the respiratory, ocular, or gastrointestinal tracts. In spite of the robust humoral and cellular immune responses found in most adults, HAdVs induce latent/persistent infections that last for decades [6]. Persistent infections are also coherent with data demonstrating that in patients undergoing pharmaceutical or disease-induced immune suppression, subsequent adenoviremia can be traced to the seroprevalence of the same type before immune suppression [6].

It is in this context that we address the impact of pre-existing humoral immunity against HAdVs on the innate immune response by primary human phagocytes. We argue that understanding the divergent roles of pDCs in antiviral immunity vs. tolerance will help resolve the discord between the robust immune response to HAdV and the ability to induce latent infections [7,8]. Our data demonstrate that pDCs take up HAdV-5 more efficiently when complexed with IgGs, the pDCs become activated via a TLR9-associated pathway, which leads to an increase in autophagy and apoptosis.

## 2. Materials and Methods

### 2.1. Ethics Statement

Blood samples from >120 anonymous donors from the local blood bank (Etablissement Français du Sang (EFS); Montpellier, France) were used during this study. All donors provided written informed consent and an internal ethics committee at IGMM and the EFS approved the study.

### 2.2. Cells and Culture Conditions 

pDCs were negatively selected from PBMCs using Plasmacytoid Dendritic Cell Isolation Kit II, human (MACS Miltenyi Biotec, Auburn, CA, USA) and cultured in RPMI 1640 medium (Cellgro, Swedesboro, NJ, USA) supplemented with 10% FBS, 10 mM HEPES, 100 units/mL penicillin, non-essential amino acids, and 1 mM sodium pyruvate at 37 °C/5% CO_2_.

### 2.3. HAdV Vectors & Hexon Peptides

Ad*β*gal is a ΔE1/E3 HAdV5 harbouring a *lacZ* expression cassette [9]. Ad^L40Q^ is an HAdV5-based vector with a leucine to glutamine mutation of an amino acid in protein VI that decreases its membrane lytic activity [10]. Alexa555- and Alexa488-HAdV5 were generated from Adβgal using an Alexa555 or Alexa488 Protein Labeling Kit (Life Technologies, Villebon-sur-Yvette, France), as previously described [11]. Ad2ts1 harbours a mutation in protease and results in several unprocessed capsid proteins and a hyper-stable capsid [12]. All HAdV viruses/vectors were produced in 293 or 911 cells and purified by double banding on CsCl density gradients, as previously described [13]. Vector purity typically reaches >99%. HAdV concentrations (physical particles/mL) were determined, as previously described [14].

### 2.4. Antibodies

Anti-human CD83-FITC (^#^556910), anti-human HLA-ABC-PE (^#^555553), anti-human HLA-DR-PE (^#^555812), anti-human CD80-FITC (^#^557226), anti-human CD86-APC (^#^555660), anti-human CD123-FIT C (^#^555432), anti-human CD303-PE (^#^561697), and anti-human CD40-APC (^#^313008) were from BioLegend. (San Diego, CA, USA).

### 2.5. Pharmacological Assays

Imiquimod is a TLR7 agonist and can inducing caspase-mediated apoptosis, and inhibits adenosine signalling in some cells. While its mechanism of action is only partially understood, in pDCs, imiquimod induces TNF secretion but not a type 1 IFN production. Chloroquine is a diprotic weak base that can sequester protons and prevent endosomal acidification. ODN 2216 is a short oligonucleotide that contains unmethylated CpG dinucleotides that can be found in pathogen genomes. The short oligonucleotides prevent TLR9-mediated signalling by inhibiting TLR9 clustering. Spautin-1, a quinazolin compound, is an inhibitor of autophagy and promotes Vps34 PI 3 (phosphoinositide 3-kinases complex) degradation by blocking the activity of USP 10. Wortmannin is a non-specific covalent inhibitor of PI 3-kinases.

### 2.6. Immune Complex Formation and DC Stimulations 

pDCs (1 × 10^5^ in 200 µL of complete medium) were incubated with HAdV5 or IC-HAdV5 (or IC) (2 × 10^4^ or 4 × 10^4^ physical particles (pp)/cell, unless otherwise indicated) for the indicated times. IC-HAdV5s were generated by mixing the virus (8 × 10^9^ physical particles) with 2.5 µL of IVIg (human IgG pooled from 5000 to 10,000 donors/batch) (Baxter SAS; Guyancourt, France) for 15 min at room temperature. IVIg is used in patients with primary or acquired immunodeficiency as well as autoimmune diseases. Chloroquine (1–10 nM) or ODN 2216 (tlrl-2216) (InvivoGen, San Diego, California, USA) was added 1 or 2 h before stimulation.

### 2.7. Quantification of mRNA

Expression levels of cytokine and chemokine genes were evaluated using RT-qPCR assays. Total RNA was isolated from cells using the high pure RNA isolation Kit (Roche; Berlin, Germany) with a DNase I treatment during the purification and subsequent elution in 50 µL of RNase-free water (Qiagen, Germantown, MD, USA). Reverse transcription was performed with the superscript first-strand synthesis system (Invitrogen) using 10 µL of total RNA and random hexamers. The cDNA samples were diluted 1:20 in water and analysed in triplicate using a LightCycler 480 (Roche; Meylan, France). SYBR green PCR conditions were as follows: 95 °C for 5 min and 45 cycles of 95 °C for 15 s, 65 °C or 70 °C for 15 s, and 72 °C for 15 s, using *GAPDH* as a standard. Relative gene expression levels of each respective gene were calculated using the threshold cycle (2^−ΔΔCT^) method and normalized to *GAPDH* mRNA levels. 

### 2.8. Co-Stimulatory Protein Levels

Surface levels of CD83, MHCII, CD80, CD40, and CD86 were assessed by flow cytometry. Cell membrane integrity was assessed by collecting cells via centrifugation at 800× *g*; the cell pellets were then resuspended in PBS containing 10% FBS, propidium iodide (PI) (Sigma-Aldrich, St. Louis MO, USA), or 7-aminoactinomycin D (7AAD) (Becton-Dickinson; Franklin Lakes, NJ, USA). The cell suspension was incubated for the indicated times and analysed using a FacsCalibur flow cytometer (Becton-Dickinson, Franklin Lakes, NJ, USA) and FlowJo software (https://www.flowjo.com/, accessed on 14 July 2021).

### 2.9. Annexin V Staining

The proportion of apoptotic thymocytes was measured by flow cytometry by staining cells for DNA using propidium iodide (PI) and surface expression of phosphatidylserine using Annexin V-FITC (Invitrogen, Waltham, MA, USA).

### 2.10. Cytokine Secretion: ELISA

Supernatant from the cells were collected and cytokine secretion was measured by ELISA and Luminex assays. The secretion of TNF and IFN-α was quantified by ELISA using an OptEIA human TNF ELISA Kit (Becton Dickinson) and human IFN-α ELISA (R&D Systems; Lille, France) following the manufacturer’s instructions. 

### 2.11. Statistical Analyses

All experiments were performed at least in duplicate a minimum of three independent times, and the results are expressed as the mean ± SD unless otherwise stated. The statistical analyses were performed using the Student’s *t*-test unless otherwise stated. A *p* value < 0.05 is denoted as significant. Statistical analyses of the global cytokine profiles (pie chart) were performed by partial permutation tests using the SPICE software. 

### 2.12. Data Availability

All data generated or analysed during this study are included in this published article.

## 3. Results

### 3.1. pDCs Take up IgG-Complexed HAdV5 

**Figure 1:**
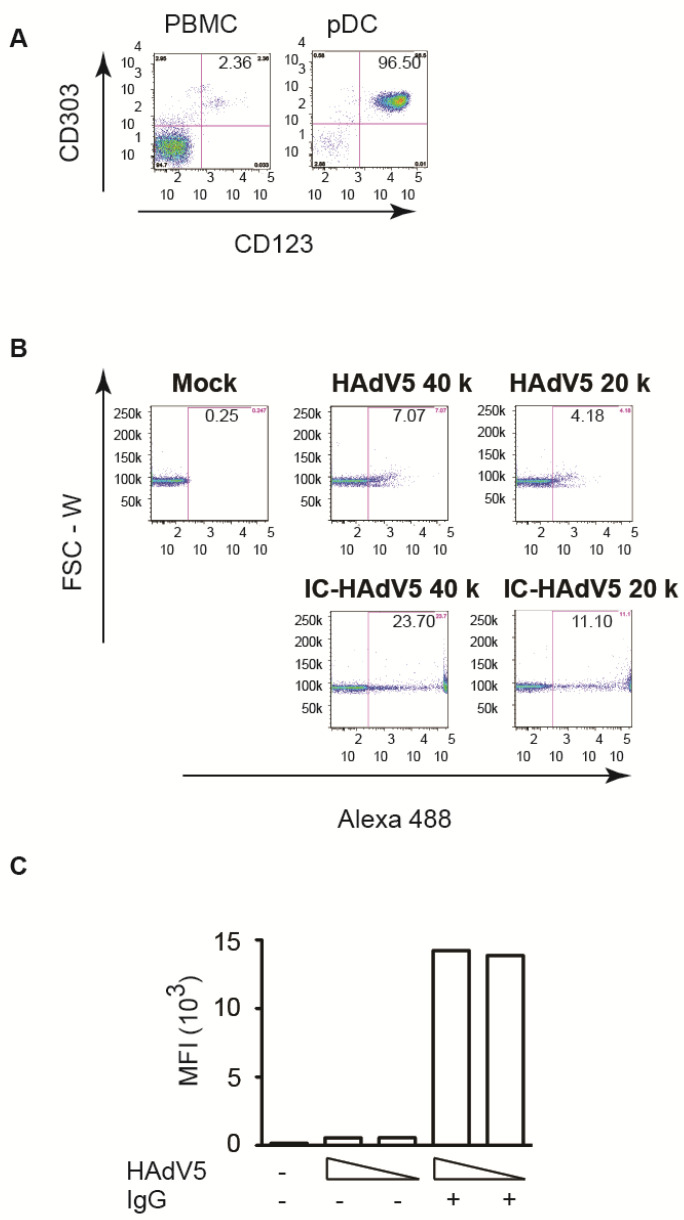
pDC purity and infection by HAdV5 and IC-HAdV5. (**A**) pDCs were isolated from PBMC by negative selection kit. pDCs and PBMCs were stained CD123 and CD303 to check pDC purity. (**B**,**C**) pDCs were challenged with IgG, HAdV5-Alexa488, or IC-HAdV5-Alexa488 at 20,000 and 40,000 pp/cell) (an example from a single donor). (**C**) Cumulative data from 3 donors. Median fluorescent intensity (MFI).

pDCs were isolated from buffy coats from random blood bank donors. The purity (>95%) and phenotype were controlled by flow cytometry using anti-CD303 and anti-CD123 antibodies (Figure 1A). Loré et al. [15] previously reported that HAdV5 vectors poorly infect pDCs. Our results are consistent with these findings: at 20,000 physical particles (pp)/cell only ~4% of the pDCs took up HAdV5 (Figure 1B). We therefore asked if the neutralizing antibodies (NAbs) in IVIg modified HAdV5 uptake. In addition to an ~3-fold increase in the number of pDCs that took up HAdV5 (Figure 1B), a striking result was the >10^4^-fold increase in fluorescence (Figure 1C). 

### 3.2. pDC Activation by IgG-Complexed HAdV5 

**Figure 2:**
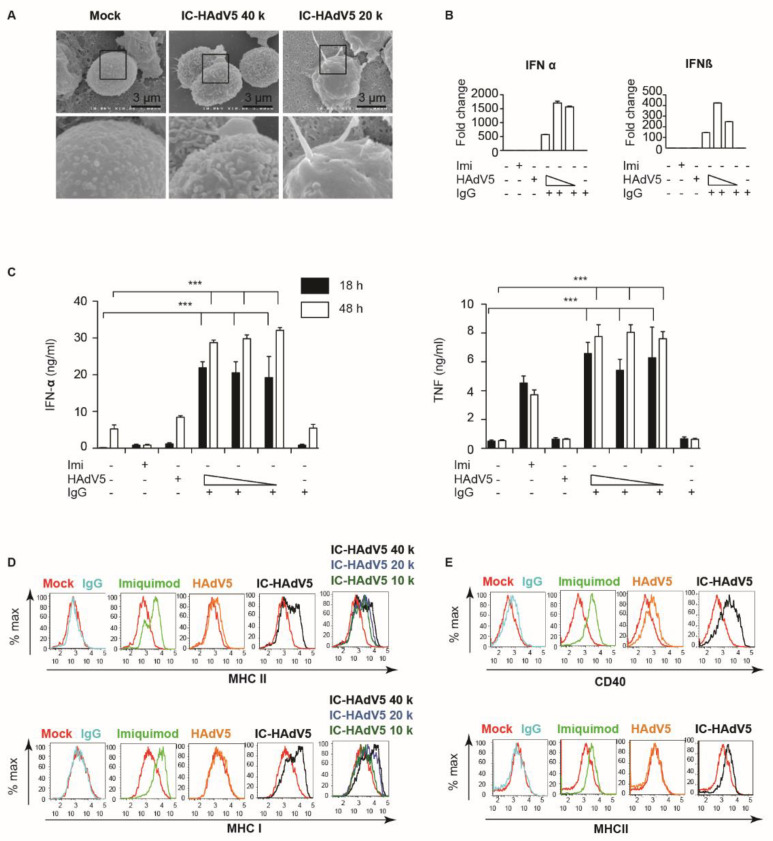
pDC activation. (**A**) Electron micrograph of pDCs mock-treated or challenged with IC-HAdV5 2—4 × 10^5^ pp/cell. Sixty cells were imaged in three independent experiment and representative images, as illustrated. (**B**) The mRNA levels of *IFNα* and *IFNβ* from pDCs exposed to the milieu from IgG, HAdV5-, and IC-HAdV-challenged at 4 h post-activation by qPCR. The cytokine sequences were designed in-house. (**C**) The IFN-α and TNF levels in the supernatant of pDCs challenged with IgG, HAdV5-, and IC-HAdV. Challenged at 18 h and 48 h post-activation. *n* ≥ 3 donors. *p* values were derived from a one-way ANOVA with Dunnett’s post-test. *** corresponds to *p* < 0.0001 and denotes significant difference. Imiquimod was used as a positive control. (**D**,**E**) The cell surface levels of MHC I and II (**D**) and CD40 (**E**). Quantified 18 h post-stimulation with Imiquimod, HAdV5, or increasing doses of IC-HAdV5. *n* = 3 donors with similar results.

Increased uptake of IC-HAdV5 by primary human monocyte-derived DCs induces phenotypic and functional maturation [8,13,16,17,18]. The changes in phenotype include dendritic morphology. We therefore qualitatively examined pDC morphology by scanning electron microscopy (SEM). Compared to mock- and HAdV5-treated pDCs, the majority of cells in the IC-HAdV5-challenged wells had larger and more complex processes (Figure 2A). We then focused on markers of activation. A prerequisite for increased cytokine secretion is the transcriptional upregulation of the respective genes. We therefore quantified *IFNα* and *IFNβ* mRNA levels in IC-HAdV5-challenged pDCs and found >100-fold increases compared to controls (Figure 2B). The increase in mRNA levels was also mirrored by an IC-HAdV5-induced increase (*p* < 0.05) in IFN-α and TNF secretion at 18 and 48 h post-challenge (Figure 2C). 

As pDCs mature, they also increase cell surface levels of activation markers. pDCs are capable of presenting both soluble and particulate exogenous antigens on both major MHC class I and II molecules [19]. We therefore used flow cytometry to determine whether the levels of CD40, and MHC I and II change when challenged by IC-HAdV5. We found that, compared to controls (IgGs, imiquimod, and HAdV5), IC-HAdV5-challenged pDCs increase the cell surface levels of all the above at 18 h post-stimulation (Figure 2D,E). Together, these data demonstrate that IC-HAdV5 induce pDC activation.

### 3.3. pH-Dependent TLR9 Engagement of IC-HAdV5

**Figure 3:**
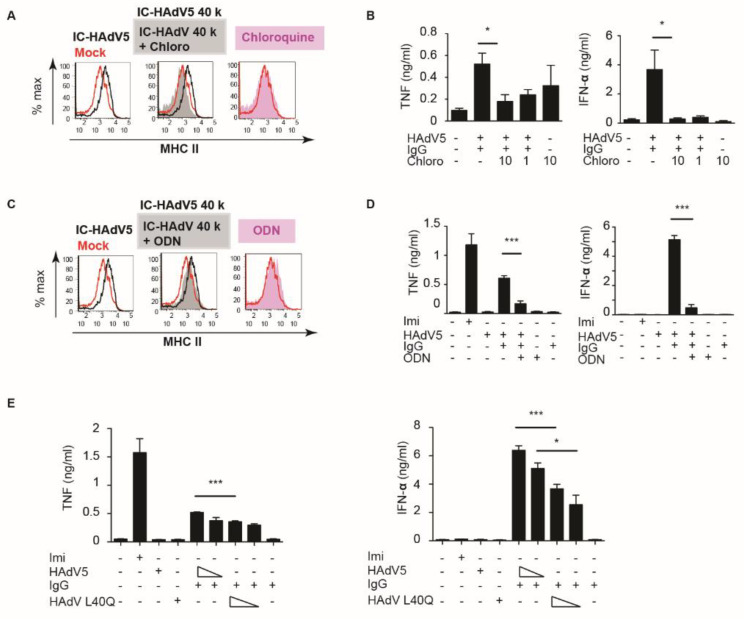
pDCs deliver IC-HAdV5 to endosomal vesicles. (**A**) pDCs were pre-treating with chloroquine (10 or 1 nM) for 2 h, then challenged with IgG, HAdV5, or IC-HAdV5. MHC II cell surface levels were quantified by flow cytometry (*n* = 3, with similar results between donors). (**B**) pDCs were pre-treating with chloroquine for 2 h, then challenged with IgG, HAdV5, or IC-HAdV5. TNF and IFN-α levels in the supernatant were quantified by ELISA (*n* = 3, *p* values were derived from *t*-test, * corresponds to *p* < 0.01). (**C**) pDC activation was assessed by pre-treating cells with 50 nM of ODN 2216 for 2 h, then stimulated with IgG, HAdV5, or IC-HAdV5. MHC II cell surface levels were quantified by flow cytometry (*n* = 3, with similar results between donors). (**D**) pDC activation was assessed by pre-treating cells with 50 nM of ODN 2216 for 2 h then stimulated with IgG, HAdV5, or IC-HAdV5. TNF and IFN-α levels in the supernatant were quantified by ELISA (*n* = 3, *p* values were derived from *t*-test, *** corresponds to *p* < 0.0001). (**E**) pDCs were challenged with IgG, HAdV5, HAdV L40Q, or IC-HAdV5; IC-HAdV L40Q in dose dependent and pDCs in each condition were collected, and detected IFN α and TNF secretion. Experiments were carried out in three independent experiments. *p* values were derived from *t*-tests. * and *** correspond to *p* < 0.01 and *p* < 0.0001, respectively.

When bound by antibodies, viruses are taken up by the Fcγ receptors expressed by numerous cells, including phagocytes [20]. It is likely that FcγR-mediated IC-HAdV5 internalization delivers HAdV components to distinct endosomal compartments for degradation, followed by presentation by MHC II and cross-presentation by MHC I molecules [21]. In contrast to prototype antigens, HAdV5 influences intracellular trafficking. As endocytic vesicles start to acidify, the metastable HAdV5 particle begins to dissociate and release protein VI [13,22]. The endosomolytic activity of protein VI allows the release of the capsid into the cytoplasm. In the context of pDCs, this process is typically slower than epithelial cells to allow efficient antigen presentation (and cross-presentation) [23]. If this were the case for IC-HAdV5, then preventing endosomal acidification should impact pDC activation. We therefore inhibited endosomal acidification with chloroquine and assayed MHC II levels, and TNF and IFN-α secretion (Figure 3A,B). Our data demonstrate that endosomal acidification was linked to pDC activation by IC-HAdV5. 

As the genomes of *Adenoviridae* are double-stranded DNA, a plausible pattern recognition receptor in pDCs to probe was TLR9 [24]. TLR9 can be inhibited using unmethylated CpG oligonucleotides (ODN 2216) sequences to prevent its oligomerization. We found that TLR9 inhibition modestly decreased MHC II levels but significantly decreased TNF and IFN-α secretion (Figure 3C,D). These data are coherent with the mechanism that the HAdV5 capsid that escapes the endocytic vesicles is partially degraded, and double-stranded DNA is available for detection by TLR9 [18,25,26].

To address this mechanism using another approach, we used AdL40Q, a HAdV5 capsid containing a mutation (L40Q) in protein VI that attenuates its membrane lytic activity [10,27,28]. Consistent with the above data, IC-AdL40Q also had a lower capacity to induce TNF, an IFN-α secretion, compared to IC-HAdV5 (Figure 3E). Together, these data demonstrate that IC-HAdV5 are taken up by pDCs in endocytic vesicles that acidify, releasing an internal HAdV5 capsid protein that then allows partially degraded capsids to be detected by TLR9, and induce pDC activation. 

### 3.4. IC-HAdV5 Induced Loss of Cell Membrane Integrity

**Figure 4:**
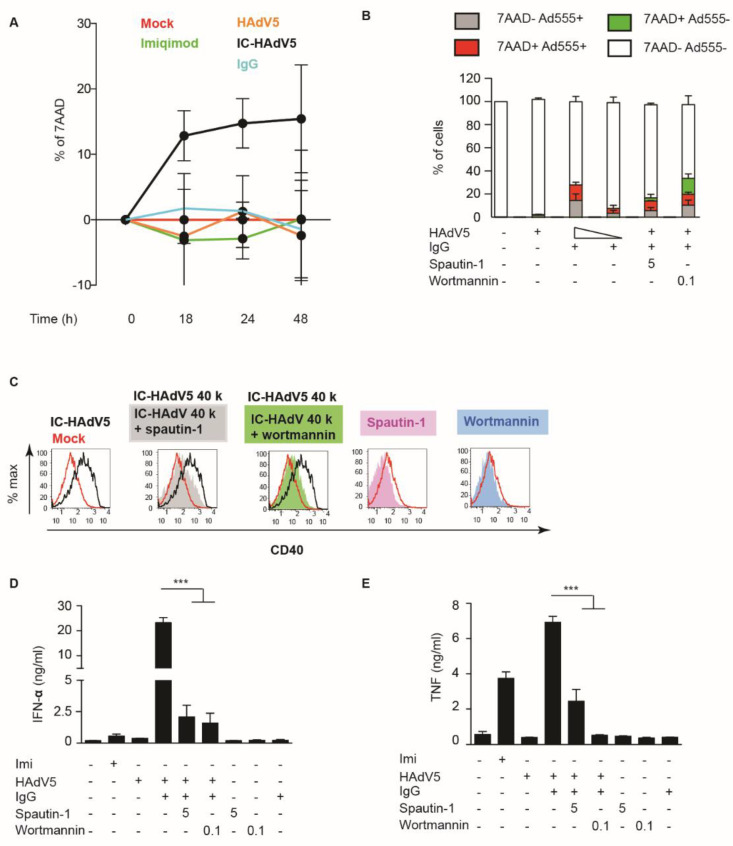
IC-HAdV5 induced loss of cell membrane integrity. (**A**) pDCs were incubated with IgG, HAdV5, and IC-HAdV5 and cell membrane integrity was assessed by intercalation of 7AAD into cellular DNA from 0 to 48 h. (**B**) pDCs were pre-treated with spautin-1 (5 μM) or wortmannin (0.1 μM) for 2 h, then were incubated with HAdV-C5-Alexa555-, IC-HAdV-555 (40,000 or 20,000 pp/cell), or IgG and assayed by flow cytometry at 18 h. The quadrants were set for 7AAD- Ad555- (white), 7AAD- Ad555 + (grey), 7AAD + Alexa555 + (red), and 7AAD + Ad555- (green). The percentage of each subpopulation at each condition is depicted in the histogram. These assays were performed in three donors with similar results. (**C**) pDCs were pre-treated with spautin-1 or wortmannin for 2 h then were incubated with IgG, HAdV5, or IC-HAdV5 in 18 h. CD40 surface expression was detected by flow cytometry. These assays were performed in three donors, with similar results. (**D**,**E**) pDCs were pre-treated with spautin-1 or wortmannin and then were incubated with IgG, HAdV5, or IC-HAdV5 in 18 h. IFN-α (**D**) and TNF secretion (**E**) were quantified by ELISA. Experiments were carried out in three donor experiments. *p* values were derived from *t*-tests. *** corresponds to *p* < 0.0001, respectively.

One of the downstream effects of IC-HAdV uptake is whether pDC survival was affected. To determine if IC-HAdVs induced the loss of plasma membrane integrity, we quantified 7AAD + entry into cells by flow cytometry. Using time-dependent assays, we found that IC-HAdV5 induce a notable increase in the number of 7AAD^+^ cells at 18 h post challenge that continued to increase (Figure 4A). To determine whether the loss of plasma membrane integrity is a direct or indirect effect of IC-HAdV5, we used IC-HAdV-555, an immune complex made using Alexa555-labeled HAdV-C5 capsid. We found that 7AAD^+^ pDCs are associated with IC-HAdV-555 (Figure 4B), suggesting that a direct interaction was responsible for the loss of plasma membrane integrity. 

**Figure 5:**
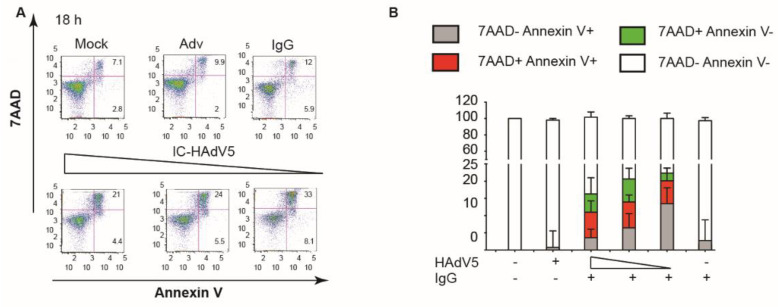
IC-HAdV5 caused autophagy and apoptosis in pDCs. (**A**) Representative flow cytometry profile of pDCs challenged with IgG, HAdV5, and IC-HAdV5 (40,000 to 10,000 pp/cell), then incubated with anti-Annexin V antibodies and assayed by flow cytometry at 18 h. (**B**) pDCs were challenged with IgG, HAdV5, and IC-HAdV5 (40,000 or 20,000 pp/cell), then incubated with Annexin V and assayed by flow cytometry at 18 h. The quadrants were set for 7AAD-/Annexin V- (white), 7AAD-/Annexin V+ (grey), 7AAD+/Annexin V+ (red), and 7AAD+/Annexin V- (green). The percentage of each subpopulation at each condition is depicted in the histogram. These assays were performed in three donors, with similar results.

We then addressed a potential synergy of autophagy and apoptosis. We used spautin-1 to increase the degradation of PI3K complexes, and wortmannin to directly inhibit PI3K activity: both inhibitors reduced pDC activation as quantified by CD40 cell surface levels (Figure 4C), and IFN-α and TNF secretion ((Figure 4D,E)**.** To address apoptosis, we incubated IC-HAdV5-challenged pDCs with 7AAD and quantified cell surface levels of Annexin V, a prototype marker of apoptosis [29]. We found that IC-HAdV5-challenged pDCs undergo apoptosis, as the majority of 7AAD^+^ cells were also Annexin V^+^ (Figure 5A,B). We did not find a notable change in Annexin V^+^ cells at the doses used. Together, these data demonstrate that IC-HAdV5 induce autophagy and apoptosis in human pDCs. 

## 4. Discussion

In this study, we explored the interaction of a human adenovirus and pre-existing humoral immunity, with primary human plasmacytoid DCs. We found that a relatively high MOI was needed to infect pDCs with HAdV5. However, pDCs more readily took up IgG-complexed HAdV5, and the susceptible cells took up more particles/cell (based on the level of transgene expression). The increased susceptibility of pDCs to IC-HAdV5 was mirrored by an increase in the morphological and functional activation. IC-HAdV5 also induced autophagy and apoptosis in pDCs. 

It would be naïve to assume that pDC purification by negative selection from human blood will generate a distinct and homogeneous population. The state-of-the-art approach to purifying pDCs from human blood may co-select a small population of pre-DCs, and therefore interpretation of our results must take this into account. pDC heterogeneity is likely linked to functional heterogeneity [30]. Typically, pDCs are resistant to most viral infections unless high doses are used. Reizis suggested that this allows them to resist virus-induced perturbations [31]. Low infection is also the case for HAdV5. The above two facts beg the question of whether the HAdV5- or IC-HAdV5-infected pDCs are a unique subset. Or, are there different subsets that are infected by HAdV5 versus those that take up IC-HAdV5? One way to address this would be intracellular staining for transcription factors (e.g., TCF4) [32] and/or IFN-1. It is not surprising that HAdV types that use CD46 as a receptor (e.g., HAdV35) more efficiently (60 fold) infect pDCs [15]. The physiological consequence of HAdV35-infected pDCs has not been examined thoroughly, and it is interesting to note that seroprevalence to HAdVs that use CD46 as a receptor (and would therefore more efficiently infect pDCs) is notably lower than HAdVs that preferentially use the coxsackievirus adenovirus receptor [33,34]. 

One question that remains to be addressed is how pDC activation is regulated and how, in the event of dysregulation, does this affect host homeostasis. pDCs play a unique role in linking innate and adaptive immune responses. Their ability to influence an adaptive immune response to viruses, via the secretion of type 1 IFN, clearly impacts the duration of infections and, paradoxically, the potential for latency. The fact that antibodies that neutralize HAdV5 infection of epithelial cells increase the uptake of pDCs is noteworthy. However, increased uptake is not unique to pDCs: conventional DCs are more susceptible to IgG-complexed HAdV infections via FcγR-mediated uptake [8,13,18,35]. While pDCs contribute to an antiviral response in a naïve host, their role in controlling or promoting HAdV persistence needs attention [36,37]. 

In our study, we focused on the initial phase of infection and did not take into account the possible impact that HAdV replication could have in pDCs secreting IFN-1. Hearing and colleagues showed that while IFNs fail to inhibit HAdV replication in cancer cell lines, replication in “normal” human cells could be inhibited by IFN-α and IFN-γ via repression of transcription of the E1A immediate early gene product. Both IFN-α and IFN-γ impede the association of E4TF-1 with the E1A enhancer region during the early phase of HAdV infection [37]. This study demonstrated that IFN signalling suppresses lytic virus replication and promotes persistent infections. Therefore, it is possible that, in some environments, IgG-complexed HAdVs are taken up by pDCs and cause the production and secretion of IFNs, which then silences the expression of HAdV genomes in bystander cells. Once transcriptionally silenced for an extended period (days?), the mechanism by which a HAdV would need to set up a “replication-friendly” environment is unknown because uptake and intracellular trafficking prime the cell for a permissive replication environment. 

Finally, in our setting, it is unknown if pDCs take up HAdV particles or genomes from other pDCs undergoing apoptosis. Viral transfer from infected cells to pDCs does occur for hepatitis C, [38], dengue, West Nile [39], and Epstein Barr viruses [40]. pDC death by the relatively quiet process of apoptosis (versus a more inflammatory death like pyroptosis [41]) could favour pathogen persistence. 

## Data Availability

All data are included in the manuscript.
